# An Attachment-Based Support in Adult Child-Parent Dyads and Parental Mental Health: A Response Surface Analysis

**DOI:** 10.1093/geroni/igaf122.1748

**Published:** 2025-12-31

**Authors:** Ella Carasso, Dikla Segel-Karpas, Shira Barzilay, Roi Estlein

**Affiliations:** University of Haifa, Cambridge, Massachusetts, United States; University of Haifa, Haifa, HaZafon, Israel; University of Haifa, Haifa, HaZafon, Israel; University of Haifa, Haifa, HaZafon, Israel

## Abstract

Intergenerational relationship between older parents and adult children offers a crucial framework for support and social belonging in older age. However, support dynamics that do not align with individual expectations and needs might have harmful effects on their mental health, causing feelings of burdensomeness and loneliness, considered significant risk factors for suicide among older adults. This study employed a dyadic attachment perspective to examine the dynamics of support exchanged between adult children and their parents, focusing on two attachment-based support forms: secure base and safe haven. We aimed to explore how the (dis)similarity in the provision of these support types between parents and adult children were associated with self-perceived burdensomeness and loneliness in older parents. A total of 129 adult child-parent dyads (parents’ age: M = 69.63; adult children’s age: M = 42.02) were recruited. We employed Structural Equation Modeling (SEM) and Response Surface Analysis (RSA) as our analytical methods. The findings show that parents experienced lower perceived-burdensomeness and loneliness when they and their children were more similar in providing both a secure base and a safe haven for each other. These results indicate that parents’ ability to rely on their children for a secure base and a safe haven promotes their mental health, when they were also able to reciprocate similar support. This emphasizes the meaningful role that parents ascribe to continuous involvement in their child’s life and the provision of support into adulthood, as well as the potential contribution of this involvement to older adults’ health.

